# Genetic Structure and Potential Environmental Determinants of Local Genetic Diversity in Japanese Honeybees (*Apis cerana japonica*)

**DOI:** 10.1371/journal.pone.0167233

**Published:** 2016-11-29

**Authors:** Teruyoshi Nagamitsu, Mika Yasuda, Fuki Saito-Morooka, Maki N. Inoue, Mio Nishiyama, Koichi Goka, Shinji Sugiura, Kaoru Maeto, Kimiko Okabe, Hisatomo Taki

**Affiliations:** 1 Hokkaido Research Center, Forestry and Forest Products Research Institute (FFPRI), Sapporo, Hokkaido, Japan; 2 Department of Forest Entomology, Forestry and Forest Products Research Institute (FFPRI), Tsukuba, Ibaraki, Japan; 3 Faculty of Science, Ibaraki University, Mito, Ibaraki, Japan; 4 Department of Applied Biological Science, Tokyo University of Agriculture and Technology, Fuchu, Tokyo, Japan; 5 Global Environment Division, National Institute of Environmental Studies (NIES), Tsukuba, Ibaraki, Japan; 6 Graduate School of Agricultural Science, Kobe University, Kobe, Hyogo, Japan; National Cheng Kung University, TAIWAN

## Abstract

Declines in honeybee populations have been a recent concern. Although causes of the declines remain unclear, environmental factors may be responsible. We focused on the potential environmental determinants of local populations of wild honeybees, *Apis cerana japonica*, in Japan. This subspecies has little genetic variation in terms of its mitochondrial DNA sequences, and genetic variations at nuclear loci are as yet unknown. We estimated the genetic structure and environmental determinants of local genetic diversity in nuclear microsatellite genotypes of fathers and mothers, inferred from workers collected at 139 sites. The genotypes of fathers and mothers showed weak isolation by distance and negligible genetic structure. The local genetic diversity was high in central Japan, decreasing toward the peripheries, and depended on the climate and land use characteristics of the sites. The local genetic diversity decreased as the annual precipitation increased, and increased as the proportion of urban and paddy field areas increased. Positive effects of natural forest area, which have also been observed in terms of forager abundance in farms, were not detected with respect to the local genetic diversity. The findings suggest that *A*. *cerana japonica* forms a single population connected by gene flow in its main distributional range, and that climate and landscape properties potentially affect its local genetic diversity.

## Introduction

Population declines in both wild and managed honeybee populations are of concern in Europe and the US [1–3], although the global population of managed honeybee colonies has increased [4]. Causes of the declines remain unclear although many factors, may be involved, such as pests and pathogens, environmental stressors (malnutrition, apicultural mismanagement, and exposure to agrochemicals and genetically modified crops), changes in cultural practices and land use, and a lack of genetic diversity and vitality [1,2].

Local abundance of honeybees depends on environmental factors. A meta-analysis was performed to determine the effects of human activities, such as grazing, logging, and agriculture, on bee abundance in accordance with habitat loss [5]. This analysis revealed that the activities did not affect the abundance of honeybees, but did decrease the abundance of other bees, including bumblebees. Despite the neutral effects of human activities on the abundance of honeybees in general, the forager density of *Apis mellifera* Linnaeus in *Brassica* fields was positively associated with the area of woody vegetation around the fields [6]. In *Apis cerana* Fabricius, visitation frequency to buckwheat flowers in farms was positively correlated with the area of forest cover within 3-km radii of the farms [7]. When natural forests and artificial plantations were discriminated, natural forests were found to have more positive effects on visitation frequency [8]. These findings suggest that the abundance of honeybees depends on the natural forest area, which provides their nest sites and floral resources.

Managed honeybees are not amenable to evaluations of the effects of environmental factors on their populations, because apicultural management affects their abundance. Although wild honeybee populations may depend on their surrounding environment, it is difficult to measure population sizes in the field. Surveying wild *A*. *mellifera* colonies requires great effort because their nests, in hollow trees or earth cavities, are difficult to detect [9]. A genetic population consists of reproductive individuals, queens and siring drones. Drones collected from a mating aggregation site, or drone congregation area (DCA), are useful for inferring the genotypes of their queens [10]. Workers collected from a colony can be used to infer the genotypes of their queen and multiple siring drones [11]. Compositions of the siring drones are likely to indicate the diversity of mates at a DCA. Assemblages of queens, inferred from foraging workers collected at a flower patch, appear indicative of the diversity of the colonies that are using the foraging patch. These diversity indices reflect the size and genetic diversity of the local population, which may in turn depend on environmental conditions and landscape properties.

A macro-ecological approach to studying wild honeybees is effective for revealing the effects of environmental factors on their population size and genetic diversity. We focused on *A*. *cerana* distributed across southern, southeastern, and eastern Asia [12]. This species belongs to the same subgenus as *A*. *mellifera*, and both are cavity-nesting species [12]. *Apis cerana japonica* Radoszkowski, distributed in Japan, is one of four subspecies of *A*. *cerana* [13]. This subspecies is present on the Japanese main islands, Honshu, Shikoku, and Kyushu, and their adjacent islands, but is absent from the northernmost island, Hokkaido, and the southernmost Ryukyu and Ogasawara Islands [14]. Pollen utilization was shown to be similar between *A*. *mellifera* and *A*. *cerana japonica* in a forest habitat in Japan [15]. Thus, nesting and foraging responses to various environmental factors seem to be similar between the two species.

The genetic structure of *A*. *cerana japonica* is known to be relatively homogeneous in its main distributional range. Thus, local genetic diversity can be compared among various locations without the confounder of geographic patterns in genetic structure. At a mitochondrial locus, a single allele is predominant in *A*. *cerana japonica* throughout Japan, although a few different alleles have been found in the northern region of the island of Honshu [16], and in the southern, adjacent islands of Tsushima and Amami-Oshima [14]. The predominant allele in Japan is the same as that found in Korea and Russia, but is different from that found in Taiwan [14,16], in which another subspecies, *Apis cerana cerana*, is distributed. The findings regarding the mitochondrial locus in *A*. *cerana japonica* suggest little genetic variation, a close relationship with Korean and Russian *A*. *cerana cerana*, and the presence of unique alleles in peripheral Japan. Genetic variations at nuclear loci have not yet been determined, although highly polymorphic nuclear loci, such as microsatellites or simple sequence repeats (SSRs), are available [17]. SSRs are though to be evolutionary neutral and to reflect population demography, such as population size. Thus, local genetic diversity in SSRs is expected to depend on environmental factors that affect local abundance.

To reveal the effects of environmental properties on local genetic diversity, we investigated *A*. *cerana japonica* genotypes at many sites in its main distributional range, at which we also measured geographical, topographical, climatic, and land use properties. First, we inferred multilocus SSR genotypes of siring drones (fathers) and queens (mothers) from workers (offspring) collected at the sites, to estimate the genetic structure of *A*. *cerana japonica*. Next, to evaluate local genetic diversity, we measured the diversity of individuals and the heterozygosity of alleles, in the inferred fathers and mothers from each site. Finally, we estimated the effects of environmental factors on the diversity and heterozygosity to elucidate the potential environmental determinants of local genetic diversity in *A*. *cerana japonica*.

## Materials and Methods

### Worker collection

**Fig 1:**
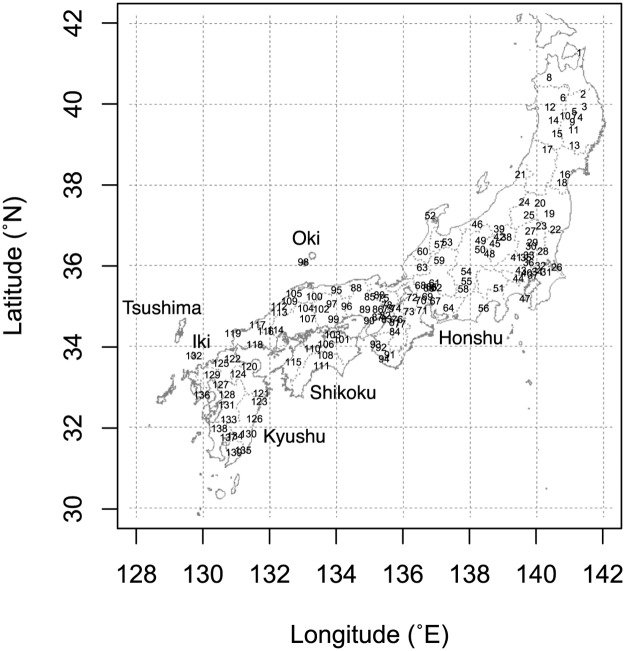
Locations of sites (*numbers*) where *Apis cerana japonica* workers were collected. Borders (*broken lines*) between prefectures are shown.

We collected *A*. *cerana japonica* workers from 139 sites on the Japanese Archipelago, including the islands of Honshu, Shikoku, Kyushu, Oki, and Iki, located between 31.398–41.266° N, 129.724–141.448° E, and 0–967 m above mean sea level (Fig 1). We selected these sites to cover various environmental conditions and landscape properties representative of the main distributional range of *A*. *cerana japonica*. We omitted the southernmost and westernmost islands, Amami-Oshima and Tsushima, respectively, where unique mitochondrial alleles have been found [14].

We collected 20 workers departing from and returning to a nest of a colony at each of 76 sites, and 20 workers foraging on a flower patch at each of 63 sites, within 1 hour, from October 2011 to September 2012 (S1 Table). The flower patch was a continuous area containing flowering herbs and shrubs within a 50-m diameter. We stored the collected workers at –20°C until DNA extraction. The DNA was extracted from a single leg of a worker using the Nonidet method [18].

### Ethics statement

The 139 sites were not in any national parks or protected areas. *Apis cerana japonica* was not assigned to an endangered or protected species in any sites, where worker bees were collected. Also, in Japan, no specific permissions are required to collect non-protected insects in non-protected area. When we collected workers from colonies kept by beekeepers or land owners, we obtained their permissions.

### Genotyping workers and inferring father and mother genotypes

We examined 17 codominant loci of nuclear SSRs, which were amplified using 12 pairs of primers designed for *A*. *cerana*, Ac1, Ac2, Ac3, Ac5, Ac11, Ac21, Ac26, Ac27, Ac30, Ac32, Ac34, and Ac35 [17], and five pairs of primers designed for *A*. *mellifera*, Ap049, Ap226, Ap243, Ap274, and Ap306 [19]. We performed polymerase chain reactions (PCRs) using a forward primer labeled with fluorescent dye at the 5'-end (FAM, NED, VIC, or PET; Life Technologies, Foster City, CA, USA) and a reverse primer assigned to each locus. PCRs were performed in 12-μL reaction mixtures containing 1.25 μL GeneAmp 10× PCR Buffer II (Life Technologies), 1.0 mM dNTP Mix, 2.1 mM MgCl_2_, 0.52 μM of each primer, 0.5 U AmpliTaq Gold DNA Polymerase (Life Technologies), and 0.25 μL template DNA solution using a TaKaRa TP600/TP650 thermal cycler (TaKaRa Bio Inc., Tokyo, Japan). A PCR process consisted of an initial 9 min at 95°C, followed by 35 cycles of 30 s at 94°C, 30 s at a primer-specific annealing temperature, 30 s at 72°C, and a final 10 min at 72°C. We applied the economic method to Ac2 and Ac3 using the universal M13 (–21) primer labeled with fluorescent dye [20]. Sizes of the PCR products were measured using an ABI PRISM 3730 Genetic Analyzer and GeneMapper analysis software ver. 3.7 (Life Technologies). Based on the product sizes, we determined the diploid genotypes of the collected workers. Because most loci were monomorphic, we obtained informative genotypes at six polymorphic loci, Ac2, Ac3, Ac27, Ac30, Ac35, and Ap049 (S2 Table).

We inferred genotypes of fathers and mothers from the worker genotypes using COLONY software ver. 2.1 [21] for haplodiploid species. In honeybees, virgin queens mate with multiple drones, while drones mate just once and then die [22]. Thus, we postulated maternal polygamy and paternal monogamy. Assuming that workers collected at each nest belonged to the same maternal sibship, we inferred the diploid genotype of their mother, and the haploid genotypes of their fathers, from the diploid genotypes of the workers. From workers collected at each flower patch, we inferred the genotypes of their parents. We did not use the priors of paternal and maternal sibship size. The rates of allelic dropout and genotyping error were both 0.001 at each locus.

### Population genetic parameters of inferred fathers and mothers

To sample reproductive individuals from the main distributional range, we selected the two most frequent haploid genotypes of fathers, and the most frequent diploid genotype of mothers, which were inferred from workers collected at each of the 139 sites. We estimated population genetic parameters for 278 haploid genotypes and 139 diploid genotypes, representing the fathers and mothers, respectively, using GenePop software ver. 4.2 [23]. First, we examined the linkage disequilibrium among the six loci in each of the haploid and diploid populations. In multiple tests of 15 pairs of loci, the statistical significance level was adjusted by the Bonferroni method. Second, we estimated the expected heterozygosity (within population: *H*_E_) in the haploid population and estimated the observed heterozygosity (within individual: *H*_O_), *H*_E_, and the fixation index (*F*_IS_ = (*H*_E_ − *H*_O_)/ *H*_E_) in the diploid population at each locus (Table 1). We also examined the deviation from Hardy-Weinberg equilibrium in the diploid population. Third, we examined isolation by distance among the sites in each of the haploid and diploid populations with the Mantel test, and estimated a regression slope from the log-transformed spatial distance (km) to the genetic distance (*F*/(1 − *F*)).

**Table 1:** Genetic variation in father and mother populations inferred from *Apis cerana japonica* workers at each locus and at multiple loci.

Locus	Father*	Mother**		
	Heterozygosity within population (*H*_E_)	Heterozygosity within individual (*H*_O_)	Heterozygosity within population (*H*_E_)	Fixation index (*F*_IS_)
Ac2	0.571	0.604	0.552	-0.094
Ac27	0.356	0.396	0.414	0.045
Ac3	0.653	0.676	0.632	-0.071
Ac30	0.613	0.669	0.632	-0.058
Ac35	0.695	0.532	0.690	0.228
Ap049	0.295	0.360	0.327	-0.101
Multilocus	0.530	0.540	0.541	0.003

*Father population consists of 278 haploids (two frequent genotypes in each site).

**Mother population consists of 139 diploids (one frequent genotypes in each site).

We performed Bayesian clustering of genotypes in each of the haploid and diploid populations using STRUCTURE software ver. 2.3.1 [24] with 20 independent runs for each number of clusters from 1 to 6. Each run included a burn-in length of 10,000 iterations and a sampling length of 100,000 iterations. Clusters were assumed to be under Hardy-Weinberg equilibrium, and both correlated allele frequency and asymmetric admixture were assumed. We regarded the number of clusters, for which the mean log-likelihood of the runs was highest, as the most plausible. We obtained allele frequencies for each cluster and examined its heterozygosity (*H*_S_) and genetic differentiation (*F*_ST_ = (*H*_T_ − *H*_S_)/ *H*_T_) from the base population with the heterozygosity (*H*_T_). We drew bar plots of the inferred ancestry of the clusters in the haploids and diploids.

### Prediction of diversity and heterozygosity from site properties

We recorded the environmental characteristics, such as geography, topography, climate, and land use, at each site. We also recorded the latitudes (°N) and longitudes (°E) of the sites as their geographical properties. Since the latitudes tended to be correlated with climatic properties, we selected longitude (*o*°E) as the geographical property. Because local genetic diversity was high in central Japan and decreased in peripheral regions (see Results), we calculated the longitudinal periphery (*h =* |*o* − ∑*o*/139|). For topographical properties, we obtained the altitude (*a* m) and the angle and direction of the slope. The mean slope angle (*g*°) and direction of the steepest slope (*d*°, north: 0°) were calculated from a digital elevation model with 10-m grids within a 250-m mesh [25]. The direction was transformed to the south-facing index (*s* = –cos(π*d*/180)) with a trigonometric function. For climatic properties, we obtained the annual precipitation (*p* mm), annual mean of the daily mean temperature (*t*°C), annual maximum snow depth (*w* cm), and annual mean daily cumulative solar radiation (*l* MJm^-2^) by interpolating 1-km mesh data from the mean values of the past 30 years [25]. For land-use properties, we measured the areas (km^2^) of paddy field (*y*), farm (*f*), urban area (*u*), natural forest (*n*), artificial forest (*c*), and grassland (*r*) within 1, 2, 3, 4, and 5-km radii of the sites based on 1:50,000 digital vegetation maps [26] using ArcGIS software ver. 10.2 (ESRI, Redlands, CA, USA). In addition to the area, we calculated the diversity of land use, with Simpson’s diversity index (*v* = 1 − ∑*e*^2^), from the proportion (0 ≤ *e* ≤ 1) of the above six land use categories and three additional categories (orchard, bamboo shrub, and wetland) to assess the complexity of land use.

In addition to the properties of the sites, we considered collection month (*m* from January: 1 to December: 12) to discriminate confounding effects of seasonality. In Japan, foraging and reproduction in honeybees appear to be most prevalent in June and September, with less activity occurring in July and August due to extremely high temperatures and shortages of floral resources [15]. In winter, from December to March, honeybees are usually inactive due to low temperatures. Thus, we set two different phases, with maxima and minima in June and December and September and March, respectively, and obtained the former (*j* = cos(π(*m* − 6)/6)) and latter (*b* = cos(π(*m* − 9)/6)) values with trigonometric functions.

We evaluated local genetic diversity as a response variable based on the diversity of parents and the heterozygosity of alleles of the parents at each of the 139 sites. We defined diversity as the proportion by which two randomly sampled individuals differed, and defined the heterozygosity as the proportion by which two randomly sampled alleles differed. The response variables for the diversity of fathers that mated with a single queen were the number of pairs of workers with different (*df*_1_) and identical (*df*_2_) fathers. The response variables for the heterozygosity of alleles of the fathers were the number of pairs of different (*hf*_1_) and identical (*hf*_2_) alleles of the fathers. The response variables for the diversity of the mothers of workers foraging on a flower patch were the number of pairs of workers with different (*dm*_1_) and identical (*dm*_2_) mothers. The response variables for the heterozygosity of alleles of the mothers were the number of pairs of different (*hm*_1_) and identical (*hm*_2_) alleles of the mothers. For the response variables of the mothers, 76 sites in which workers were collected at nests were removed from the data because there was a single mother at each of these sites.

We fitted a linear model of explanatory variables, *h*_*i*_, *a*_*i*_, *g*_*i*_, *s*_*i*_, *p*_*i*_, *t*_*i*_, *w*_*i*_, *l*_*i*_, *j*_*i*_, *b*_*i*_, *y*_*ij*_, *f*_*ij*_, *u*_*ij*_, *n*_*ij*_, *c*_*ij*_, *r*_*ij*_, and *v*_*ij*_ (Table 2), for site *i* at spatial scale *j* (1, 2, 3, 4, and 5-km radii) to each set of response variables, the diversity and heterozygosity of fathers (*df*_*i*1_, *df*_*i*2_) and (*hf*_*i*1_, *hf*_*i*2_) and those of mothers (*dm*_*i*1_, *dm*_*i*2_) and (*hm*_*i*1_, *hm*_*i*2_), using glmmML (family = binomial (link = "logistic")) in the glmmML package of the R software ver. 3.1.3 [27]. The explanatory variables were standardized using scale () in R. For the heterozygosity, the model included fixed effects of loci. The model included random effects of sites to reduce the influence of over-dispersion and to evaluate the individuality of sites.

**Table 2:** Effects of environmental propertie and collection month on local genetic diversity in *Apis cerana japonica*. Coefficients for standardized explanatory variables estimated in a model with the lowest Akaike’s information criterion are shown.

Standerdized explanatory variable	Father		Mother	
	Diversity	Heterozygosity	Diversity	Heterozygosity
Spatial scale*	5 km	2 km	1 km	1 km
Longitudinal periphery (*h*)	-0.138	-0.171	-0.294	-0.094
Altitude (*a*)				0.089
Slope angle (*g*)				0.129
Slope south-facing (*s*)		0.073		
Percipitation (*p*)	-0.184	-0.105		-0.181
Mean temperature (*t*)	0.153			0.172
Maxinum snow depth (*w*)				0.126
Mean solar radiation (*l*)	0.105			
Season (max: Jun, min: Dec) (*j*)	0.291			0.123
Season (max: Sep, min: Mar) (*b*)	0.132		0.226	
Paddy field (*y*)			0.149	0.145
Farmland (*f*)				
Urban area (*u*)	0.224			0.070
Natural forest (*n*)		-0.093		
Artificial forest (*c*)				
Grassland (*r*)				
Landuse diversity (*v*)				

*Spatial scales for the land use properties are examined among 1, 2, 3, 4, and 5-km radii from each site.

We fitted the models with possible combinations of explanatory variables, at each of the five spatial scales, to each set of response variables. Then, we selected a model with the lowest Akaike’s information criterion (AIC) value, using dredge (rank = "AIC") in the MuMIn package in R. When there were too many explanatory variables to finish the model selection within 1 hour, we removed some explanatory variables, which had small effects in the full model, from the selected models. Among the selected models at the five spatial scales, we further selected a model with the lowest AIC value. In the finally selected model, we estimated the coefficient of the fixed effects and the standard deviation (SD) of the random effects. We obtained predictions from the explanatory variables using the estimated effects, particularly those with consistent effects among the four response variables.

## Results

### Genetic structure inferred for fathers and mothers

We determined the diploid genotypes of 2,738 workers from 139 sites. At the 76 sites, from which we collected workers at nests, we inferred 717 father genotypes from 1,508 worker genotypes. We also inferred, at 63 sites, from which we collected workers at flower patches, 650 father genotypes from 1,230 worker genotypes. We inferred 254 mother genotypes for each of the 63 sites, from which we collected workers at flower patches. We also inferred 76 mother genotypes, a single mother for each of the 76 sites, from which we collected workers at nests.

In a population of 278 haploids representing the inferred fathers, we found significant linkage disequilibrium only between Ac30 and Ac35 (*P* = 0.020). In a population of 139 diploids representing the inferred mothers, we found no significant linkage disequilibrium (*P* ≥ 0.190). The expected heterozygosity (*H*_E_) was 0.530 in the father population and 0.541 in the mother population (Table 1). The observed heterozygosity (*H*_O_) was 0.540, and the fixation index (*F*_IS_) was 0.003, in the mother population (Table 1). The mother population significantly deviated from Hardy-Weinberg equilibrium (*P* < 0.001). We found significant isolation by distance (*P* < 0.001) and positive regression slopes (*B*) from the log-transformed spatial distance (km) to the genetic distance (*F*/(1 − *F*)) in the father (*B* = 0.0253) and mother (*B* = 0.0356) populations.

**Fig 2:**
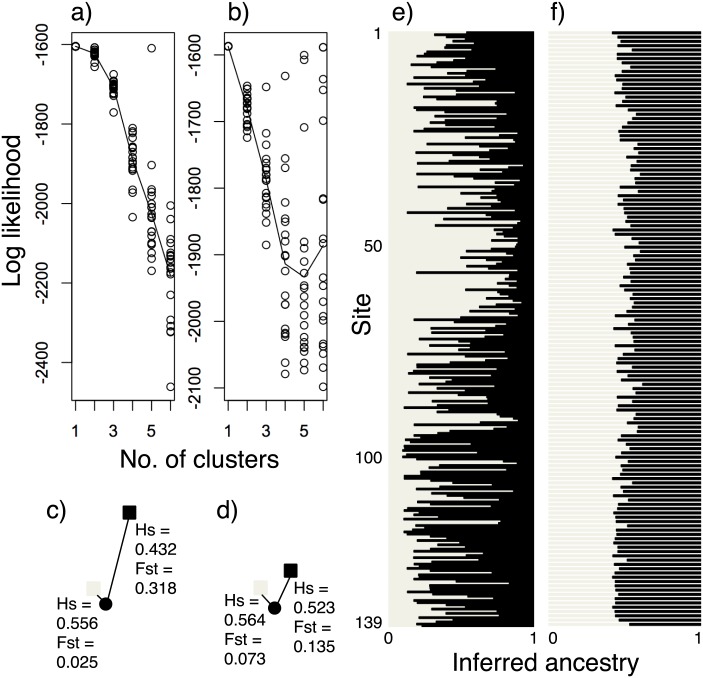
Bayesian clustering of haploid genotypes of 278 fathers and diploid genotypes of 139 mothers inferred from *Apis cerana japonica* workers. Changes in the mean log-likelihood (*lines*), and its values in each run (*circles*), according to the number of clusters of fathers (a) and mothers (b). For two clusters, genetic diversity (*H*_S_) and differentiation (*F*_ST_) of clusters 1 (*grey squares*) and 2 (*black squares*) from the base populations (*black circles*) is shown in fathers (c) and mothers (d). Bar plots of the proportion (inferred ancestry) of the two clusters (1: *grey bars*, and 2: *black bars*) for fathers (e) and mothers (f) at 139 sites.

The mean log-likelihood of Bayesian clustering was highest, when the number of clusters was one for both fathers and mothers (Fig 2a and 2b). Some runs had high log-likelihoods at multiple clusters (Fig 2a and 2b). When the number of clusters was two, the two clusters had different values for genetic diversity and differentiation from the base population (Fig 2c and 2d). Cluster 1 showed higher heterozygosity and less differentiation (*H*_S_ = 0.556, *F*_ST_ = 0.025 in the fathers; and *H*_S_ = 0.564, *F*_ST_ = 0.073 in the mothers) than cluster 2 (*H*_S_ = 0.432, *F*_ST_ = 0.318 in the fathers; and *H*_S_ = 0.523, *F*_ST_ = 0.135 in the mothers; Fig 2c and 2d). Bar plots for both fathers and mothers demonstrated that cluster 1 was slightly more frequent in central Japan, around sites 20 to 90, than in the peripheries (Fig 2e and 2f).

### Prediction of diversity and heterozygosity from environmental properties

We obtained 15 explanatory variables: one geographic (*h*_*i*_), three topographic (*a*_*i*_, *g*_*i*_, and *s*_*i*_), four climatic (*p*_*i*_, *t*_*i*_, *w*_*i*_, and *l*_*i*_), and seven land use (*y*_*ij*_, *f*_*ij*_, *u*_*ij*_, *n*_*ij*_, *c*_*ij*_, *r*_*ij*_, and *v*_*ij*_) properties, from site *i* at spatial scale *j* (1, 2, 3, 4, and 5-km radii), as well as two properties of collection month (*j*_*i*_ and *b*_*i*_; Table 2). Among the explanatory variables at the medium spatial scale (3-km radius), the Kendall’s correlation coefficients between the variables ranged from –0.56 to 0.51 for the 139 sites.

We counted the number of pairs of different (3 ≤ *df*_1_ ≤ 183) and identical (4 ≤ *df*_2_ ≤ 59) fathers that mated with a single mother, and the number of pairs of different (0 ≤ *hf*_1_ ≤ 153) and identical (8 ≤ *hf*_2_ ≤ 190) alleles of fathers, for the 139 sites. We also counted the number of pairs of different (0 ≤ *dm*_1_ ≤ 165) and identical (11 ≤ *dm*_2_ ≤ 190) mothers of workers foraging on a flower patch, and the number of pairs of different (0 ≤ *hm*_1_ ≤ 49) and identical (0 ≤ *hm*_2_ ≤ 66) alleles of mothers at each locus in the 63 sites. Based on these variables, we obtained mean data, across the six loci, on the diversity of fathers (0.272 ≤ *df*_1_/(*df*_1_ + *df*_2_) ≤ 0.964) and mothers (0.000 ≤ *dm*_1_/(*dm*_1_ + *dm*_2_) ≤ 0.869), and on the heterozygosity of alleles of fathers (0.103 ≤ *hf*_1_/(*hf*_1_ + *hf*_2_) ≤ 0.592) and mothers (0.267 ≤ *hm*_1_/(*hm*_1_ + *hm*_2_) ≤ 0.643).

The selected model with respect to the diversity of fathers included seven fixed effects: longitudinal periphery (*h*), annual precipitation (*p*), annual mean of the daily mean temperature (*t*), annual mean of daily cumulative solar radiation (*l*), two indices of collection month (*j* and *b*), and the proportion of urban area (*u*) within a 5-km radius (Table 2). Coefficients of these fixed effects of standardized explanatory variables (SD = 1) ranged from –0.184 to 0.291, while the SD of the random effects was 0.553. The selected model with respect to the heterozygosity of the alleles of fathers included the effects of loci and four fixed effects, namely *h*, the south-facing index of slope (*s*), *p*, and the area of natural forest (*n*) within a 2-km radius (Table 2). Coefficients of these fixed effects ranged from –0.171 to 0.073, while the SD of the random effects was 0.325.

The selected model with respect to the diversity of mothers included three fixed effects, namely *h*, *b*, and the area of paddy field (*y*) within a 1-km radius (Table 2). Coefficients of these fixed effects ranged from –0.294 to 0.226, while the SD of the random effects was 0.534. The selected model with respect to the heterozygosity of the alleles of the mothers included the effects of loci and nine fixed effects, namely *h*, altitude (*a*), mean angle of slope (*g*), *p*, *t*, annual maximum snow depth (*w*), *j*, and *y* and *u* within a 1-km radius (Table 2). Coefficients of these fixed effects ranged from –0.181 to 0.172, while the SD of the random effects was 0.183.

**Fig 3:**
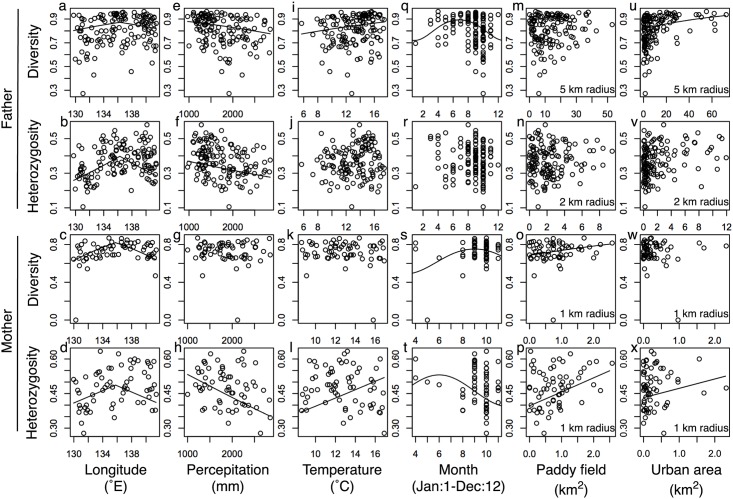
Effects of environmental properties and of collection month on local genetic diversity in *Apis cerana japonica*. Environmental properties are longitude (a, b, c, d), annual precipitation (e, f, g, h), annual mean of the daily mean temperature (i, j, k, l), and paddy field (q, r, s, t) and urban areas (u, v, w, x) at the sites. Collection month (m, n, o, p) is also shown. Diversity and heterozygosity are plotted for the fathers at 139 sites and mothers at 63 sites. Predictions (*lines*) pertaining to diversity and heterozygosity were obtained from the explanatory variables in models with the lowest Akaike’s information criterion, when other explanatory variables are given as mean values.

Seven explanatory variables, *h*, *p*, *t*, *j*, *b*, *y*, and *u*, had consistent effects on multiple response variables (Table 2). The effect of *h* was consistently negative, indicating that local genetic diversity was lower in the peripheries than in the center of the main distributional range (Fig 3a–3d). The effect of *p* was negative in three of the four response variables, indicating that local genetic diversity decreased as the annual precipitation increased (Fig 3e–3h). The effect of *t* was positive in two of the four response variables, although the patterns of these results were not clear (Fig 3i–3l). The local genetic diversity changed seasonally, with the maximum diversity occurring in the summer and the minimum diversity occurring in the winter (Fig 3m–3p). The local genetic diversity of mothers of workers foraging on a flower patch increased as the area of paddy field (*y*) increased at the 1-km radius spatial scale (Fig 3q–3t). The diversity of fathers mated with a single mother, and the heterozygosity of the alleles of the mothers of workers foraging on a flower patch, increased as the area of urban area (*u*) increased (Fig 3u–3x). The spatial scale of the selected model for the diversity of fathers was a 5-km radius, and that for the heterozygosity of maternal alleles was a 1-km radius (Table 2).

## Discussion

### Genetic structure

Geographical subdivision in genetic structure was revealed in *A*. *cerana cerana* in Thailand using nuclear SSRs [28]. Genetic structures of nuclear loci remained unknown in *A*. *cerana japonica*, although mitochondrial DNA sequences suggested little genetic variation in Japan [14]. In our study, the parameters of population genetics and the Bayesian clustering of genotypes at six nuclear SSR loci also suggest negligible genetic structure. The examined population, which consists of fathers or mothers inferred from the collected workers, covers the main distributional range of *A*. *cerana japonica* (Fig 1). The examined loci have sufficient variation (Table 1), and most are independent from the others because linkage disequilibrium was found only in a single pair of loci in the father population. Therefore, the observed genotypes are appropriate for assessing the genetic structure. The fixation index (*F*_IS_) is known to be positive in substructured populations due to the Wahlund effect [29]. The nearly zero value (*F*_IS_ = 0.003) in the mother population indicates weak substructure within that population (Table 1). However, the mother population significantly deviated from Hardy-Weinberg equilibrium, which may have resulted from a positive *F*_IS_ at Ac35 (Table 1), probably due to null alleles at this locus. Thus, the deviation does not necessarily indicate substructuring. The log-likelihood of Bayesian clustering indicates a single cluster in each population of the fathers and mothers (Fig 2a and 2b), suggesting a single random-mating population. These findings suggest negligible genetic structure in *A*. *cerana japonica* at nuclear SSR loci.

In spite of the weak genetic structure, we found isolation by distance in both the father and mother populations, indicating that genetic differentiation increases as spatial distance increases between individuals. This geographical heterogeneity may be related to the Bayesian clustering patterns of the two clusters. Cluster 1 was more frequent in central Japan (Fig 2e and 2f), and had higher genetic diversity and less differentiation from the base population, than cluster 2. In accordance with this, local genetic diversity was higher in central Japan than in the peripheries (Table 2, Fig 3a–3d). Since most nuclear SSR loci are thought to be evolutionarily neutral in spite of their potential functions under selection [30], their genetic variation seems to depend on population size and demographic history. The neutral theory predicts that larger populations have higher genetic variation. These findings suggest that the *A*. *cerana japonica* population has been larger in central Japan than in the peripheries. Generally, genetic diversity peaks in the center of a distributional range, where optimal environmental conditions may lead to the largest population size [31]. Although high genetic diversity has been frequently observed in core populations [32], genetic diversity sometimes increases in peripheral populations, in which fluctuating and diverse habitat conditions as well as hybridization with related species may increase genetic variation [33]. In managed *A*. *mellifera*, introgression often increases genetic diversity [34]. The low genetic diversity observed in the western periphery, close to the distributional range of another subspecies, *A*. *cerana cerana*, implies rare introgression from this subspecies to *A*. *cerana japonica*.

In mitochondrial DNA haplotypes, a common haplotype is prevalent throughout Japan, and some minor haplotypes that differ with a few mutations have been observed in peripheral Japan. This evidence indicates more genetic variations in peripheral populations. This pattern conflicts with our observation of more variations at nuclear SSR loci in the central populations, because more genetic variations are expected in larger populations. Thus, the origin and maintenance of the minor haplotypes in peripheral Japan requires another explanation related to the demographic history of *A*. *cerana japonica*. For instance, population expansion in central Japan has replaced the preexisting minor haplotypes with the common haplotype. Because only six nuclear SSR loci were examined in this study, it is difficult to estimate the demographic history. Information from genomic skimming using high-throughput sequencing technology is effective to reveal detailed genetic structure and population demography.

### Determinants of local genetic diversity

The density of *A*. *cerana japonica* foragers in farms was observed to increase with increasing surrounding natural forest area [6–8]. Natural forests provide nesting resources, tree hollows and earth cavities [9], and floral resources, mass-flowering trees [15]. Thus, we expected positive effects from a greater area of natural forest on local genetic diversity. In contrast to this expectation, we found positive effects of paddy field and/or urban areas on the diversity of fathers mated with a single mother and on the diversity and heterozygosity of mothers of workers foraging on a flower patch (Table 2, Fig 3q–3x). The most predictable spatial scales of these effects were radii of 5 km for the fathers and 1 km for the mothers, suggesting that honeybees disperse across larger ranges during mating compared to foraging [35,36]. These results imply that areas disturbed by human activities, rather than natural forest areas, have positive effects on local genetic diversity.

The detected effects of urban and agricultural areas on local genetic diversity should be carefully interpreted, because the examined models could not include major determinants of local genetic diversity. The SD of the random effects of sites was consistently larger than the absolute values of the fixed effect coefficients of the standardized explanatory variables (Table 2). This suggests that unknown factors, which were not included in the models, have larger effects on local genetic diversity than any of the explanatory variables. The following hypotheses can explain the unexpected results.

First, the genetically effective population size of *A*. *cerana japonica* may be larger in urban and agricultural areas than in natural forests, even if the colony size and forager population may be larger in natural forests. The diversity of fathers mated with a single mother depends on various factors, such as the availability of mates at a DCA and the mating preferences of virgin queens. The spaces above tall trees are known to be DCAs in *A*. *cerana japonica* [37]. If the density of DCAs is lower in urban and agricultural areas than in natural forests, drones from more colonies, over a larger range, aggregate at each DCA in urban and agricultural areas. Such a large mating range and high mate availability may increase effective population size and local genetic diversity.

Second, aggregation of foragers to fewer flower patches may be responsible for the observed results. If the density of flower patches is lower in urban and agricultural areas than in natural forests, foragers from more colonies, over a larger range, will visit a flower patch in urban and agricultural areas. Wild plants in natural forests tend to offer a continuous supply of floral resources throughout the season at spatially dispersed flower patches. On the other hand, cultivated plants in urban and agricultural areas, and mass-flowering crops in particular, seem to provide highly rewarding floral resources that are temporally and spatially aggregated. Honeybees preferred mass-flowering crops in farms to wild plants in semi-natural habitats [38], probably because they can allocate foragers to the most profitable resources.

The local genetic diversity at each site seems to have been estimated properly, because the geographic pattern (higher diversity in central Japan) is consistent with the observed genetic structure of samples representing individual sites (occurrence of a cluster with higher diversity in central Japan). To accurately estimate the effective population size and colony density, collection of drones at DCAs, or by using pheromone traps [39], are useful methods to infer the queen genotypes of colonies in the surrounding areas [10]. The colony density over a wide range can be estimated from these samples, because 90% of matings of *A*. *mellifera* occur within a distance of 7.5 km, and the maximum mating distance was shown to be 15 km in semi-isolated valleys [36].

Among the climatic properties, annual precipitation has negative effects on local genetic diversity (Table 2, Fig 3e–3h). Rainfall restricts opportunities for foraging and mating. Wet conditions may increase the risk of the spread of diseases and parasites, because humidity is likely to facilitate their infection and growth. In contrast to our results, rainfall positively affected Africanized *A*. *mellifera* populations in urban environments surrounded by natural desert areas, because rainfall was likely to increase floral resources in such a dry climate [40].

Local genetic diversity in the summer was higher than in the winter (Table 2, Fig 3m–3p). This pattern corresponds to seasonal changes in the foraging activities of honeybees in Japan [15]. In the summer, the allocation of workers to foragers in a colony seems to increase, and various patrilines may participate in foraging [41]. This may lead to the observed increases in the paternal diversity of the foragers. The foraging activity of a colony also tends to increase in the summer, which may result in the observed increases in the maternal diversity of foragers at a flower patch.

### Implications for conservation and management of *A*. *cerana japonica*

Population declines in honeybees have been a recent concern in Europe and the US, and conservation and management of wild honeybees is necessary [2,3]. While it is difficult to measure honeybee populations directly in the field, genetic analyses are useful for elucidating the implications for the conservation and management of honeybees. One such implication is the designation of evolutionary significant units or conservation units [42]. Our observation of the genetic structure suggests that *A*. *cerana japonica* forms a single population connected by gene flow across the Japanese main islands. Thus, the *A*. *cerana japonica* population can be treated as a single management unit.

Our study provides no evidence of positive effects of natural forests on local genetic diversity, even though forager abundance in farms is known to increase with increasing area of surrounding natural forests [6–8]. In contrast, urban and agricultural land use, where environmental stressors such as agrochemicals and unsuitable habitat conditions are thought to be common, seem to have positive effects on local genetic diversity. Further studies are necessary to reconcile these inconsistent findings and to elucidate the mechanisms underlying the effects of different environmental factors on honeybee populations. This knowledge will be useful for the management and conservation of wild honeybees in various landscapes.

## Supporting Information

S1 Table Properties of the 139 sites, from which *Apis cerana japonica* workers were collected. (TXT)

S2 Table Genotypes of *Apis cerana japonica* workers collected from 139 sites. (TXT)
